# Elastic Nanoparticle‐Reinforced, Conductive Structural Color Hydrogel With Super Stretchability, Self‐Adhesion, Self‐Healing as Electrical/Optical Dual‐Responsive Visual Electronic Skins

**DOI:** 10.1002/EXP.70008

**Published:** 2025-02-04

**Authors:** Min Xu, Junlong Liao, Jiajia Li, Yu Shi, Ziyu Zhang, Yifu Fu, Zhongze Gu, Hua Xu

**Affiliations:** ^1^ State Key Laboratory of Digital Medical Engineering School of Biological Science and Medical Engineering Southeast University Nanjing China; ^2^ Institute of Biomedical Devices (Suzhou) Southeast University Nanjing China

**Keywords:** conductive hydrogel, mechanochromic, photonic crystals

## Abstract

Developing smart hydrogel with excellent physicochemical properties and multiple signal output capability for interactively electronic skin still remains challenging. Here, conductive structural color hydrogels with desirable physicochemical properties (including high stretchability and robustness, self‐adhesion and self‐healing) were developed to provide synchronous electronic and visual color signals for e‐skins. Highly charged elastic nanoparticles were elaborately used as building units for structural color and the hydrogel were prepared by the self‐assembly of the nanoparticle to form a non‐close‐packed array in a mixture comprised of acrylamide, silkworm silk fiber proteins (SF), reduced graphene oxide (rGO) and then photopolymerization. Benefiting from the improved interfacial compatibility between flexible hydrogel network and elastic nanoparticle, covalent cross‐linking network structure and synergistic multiple non‐covalent bonding interactions, the hydrogel exhibits extraordinary mechanical properties, excellent self‐adhesion to diverse substrates and self‐healing at room temperature. In addition, the hydrogel exhibited sensitive resistance changes and synchronous structural color changes under strain. As a proof‐to‐concept, the hydrogel displayed superior capability for the color‐response and the electrical signal response of various human motions, the spatial distribution of external mechanical stimuli as well as identification of different external stimuli, indicating promising applications in the fields of interactive visual electronic skin, wearable devices, and human–machine interfaces.

## Graphical abstract

A conductive structural color hydrogel with highly charged elastic nanoparticles was developed by implementing a simple one‐step assembly and photopolymerization strategy that can achieve excellent mechanical robustness, self‐adhesiveness, self‐healing properties, as well as synchronous electronic and visual signal monitoring for interactively visual electronic skins.

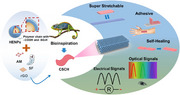

## 1 Introduction

In recent decades, artificial electronic skins (e‐skins) that can mimic human skin sensory functions have gained increasing attention for their revolutionary applications in the fields of motion and health monitoring, disease diagnosis, implantable devices, soft robots, and so on [1]. Great effort has been directed towards developing novel materials with high performance for use in e‐skins, among which hydrogels are regarded as one of the most ideal candidates due to their excellent biocompatibility, high flexibility, and elastic modulus similar to that of the human skin (≈0.5–500 kPa) [2]. Typically, hydrogels are usually integrated with conductive materials such as ions, conductive polymers, or carbon‐based nanomaterials to enhance the electronic performance of the hydrogel matrix, whereas the detective principle lies in the conversion of external stimulus into electrical signals [3]. Although significant progress has been made in the fabrication of conductive hydrogel e‐skins, the present sensing mode by a single electrical signal might not be reliable in complex environments and cannot satisfy the increasing demands of visual human–machine interaction.

In nature, some organisms (e.g., chameleons, beetle shells, and butterfly wings) can employ their skin as an interactive interface, which responds to external stimuli by adjusting the lattice spacing of guanine nanocrystal arrays to achieve changes in the skin color [4]. Drawing inspiration from these biological systems, integrating conductive hydrogels with photonic crystal structural color has been considered a promising strategy to provide synchronous electronic and visual color signals for e‐skins [5]. Some conductive structural color hydrogels have been already reported in the literature for the development of interactively visual e‐skin, in which the electrical signals offer precise sensing capability, while the color changes provide visual information and direct intelligent interaction with the human body [6].

To fit well with e‐skins and improve their detection durability and stability, conductive structural color hydrogels are typically endowed with excellent adhesive and self‐healing properties. Excellent adhesion ensures a secure fit at interfaces, enabling adaptation to complex topological surfaces and dynamic deformation processes. This effectively avoids delamination and friction thereby achieving stable and reliable sensing [7]. Meanwhile, healable gels can repair themselves in response to damage and recover their original structures and properties, which improves their reliability and service life [8]. Therefore, the development of conductive structural color hydrogels with excellent adhesion, self‐healing, and stretchable capabilities, as well as synchronous electronic and visual signal features, is highly desired for e‐skin applications. Sun et al. developed a conductive structural color hydrogel with a three‐layer structure by introducing an adhesive polyzwitterionic gel network on opal photonic crystals, which demonstrated outputting synergistic electrical and optical signals under strain load with robust adhesion, admirable stability, and high resilience [9]. Zhao and Lai respectively reported a conductive inverse opal hydrogel by integrating the adhesive polydopamine gel layer onto an inverse opal scaffold, which showed stable stretchability and high tissue adhesiveness, capable of adhering to human skin as a dual‐signal sensor for real‐time color sensing and electronic signal monitoring [10]. Although several strategies have been proposed for the preparation of functional conductive structural color hydrogels, some key issues are still unresolved: (1) The present conductive structural color hydrogels all used a multilayer structure to achieve the dual‐signal detection and adhesion function, in which the adhesive conductive gel layer was integrated on the opal/inverse opal layer to form a double‐/three‐layer structure. However, the different layer structure usually suffers from unsuitable matching and even delamination, resulting in poor structure stability and robustness, limited stretchability, lack of healing properties, and low response to external stimuli; (2) Although SiO_2_ nanoparticles have been used extensively as building units for construction of structural color hydrogels, their hard structure is difficult to be compatible with the flexible hydrogel network, which could negatively affect their mechanical stretchability, stability, and durability; (3) The fabrication process is complicated and time‐consuming, and usually requires a self‐assembly photonic crystal as template, multiple transfer, and photopolymerization on the template and removal of the template for inverse opal. These issues limit the further development and application of conductive structural color hydrogels for visual interactive e‐skins. In particular, to the best of our knowledge, high‐performance conductive structural color hydrogels with multifunctionality (e.g., high mechanical properties, adhesion and healing properties, and wide optical sensing range) are still rare. It is imperative that novel hydrogels for visually interactive e‐skins require breakthroughs in material design and fabrication.

Along these lines, a unique conductive structural color hydrogel (named CSCH) with a one‐layer covalent cross‐linking network structure was developed in this work by implementing a simple one‐step assembly and photopolymerization strategy that can achieve excellent mechanical robustness, self‐adhesiveness, self‐healing properties, as well as synchronous electronic and visual signal monitoring. Specifically, highly charged elastic poly(methyl methacrylate‐butyl acrylate) copolymer nanoparticles (HENPs) were elaborately applied as building units for structural color and improved the interfacial compatibility with the flexible hydrogel network composed of polyacrylamide (PAM) and SF by introducing multiple interfacial noncovalent interactions, while rGO was introduced to endow the hydrogel with conductivity. Benefiting from its improved interfacial compatibility and one‐layer covalent cross‐linking network structure, the hydrogel exhibited extraordinary mechanical properties with a maximum tensile strength of 0.25 MPa, elongation at break over 950%, compressive stress over 0.3 MPa, and Young's modulus at approximately 16.3 kPa. Furthermore, a large number of multiple hydrogen‐bonding and dipole–dipole interaction endow the hydrogel with excellent self‐adhesion and self‐healable properties. The resultant hydrogels exhibited sensitive resistance changes and synchronous structural color changes (gauge factor [GF]: 1.46 for 0%–100% and 3.31 for 100%–400%; Δ*λ* > 206 nm for 0%–160%) under the application of strain loads. More attractively, the fabricated CSCH film can adhere to the skin without other fixtures for color‐response and electrical signal response of various human motions and spatial distribution of external mechanical stimuli, as well as identification of different external stimuli, such as normal pressure and shear force. Compared to the reported CSCH, the proposed CSCH has comparative advantages of using a layer structure to achieve self‐adhesive and synchronous electronic and visual signal monitoring, as well as excellent mechanical robustness, self‐healing properties and simple, low‐cost, and easy to mass‐producing characteristics.

## 2 Result and Discussion

### 2.1 Design and Fabrication of CSCH

**FIGURE 1:**
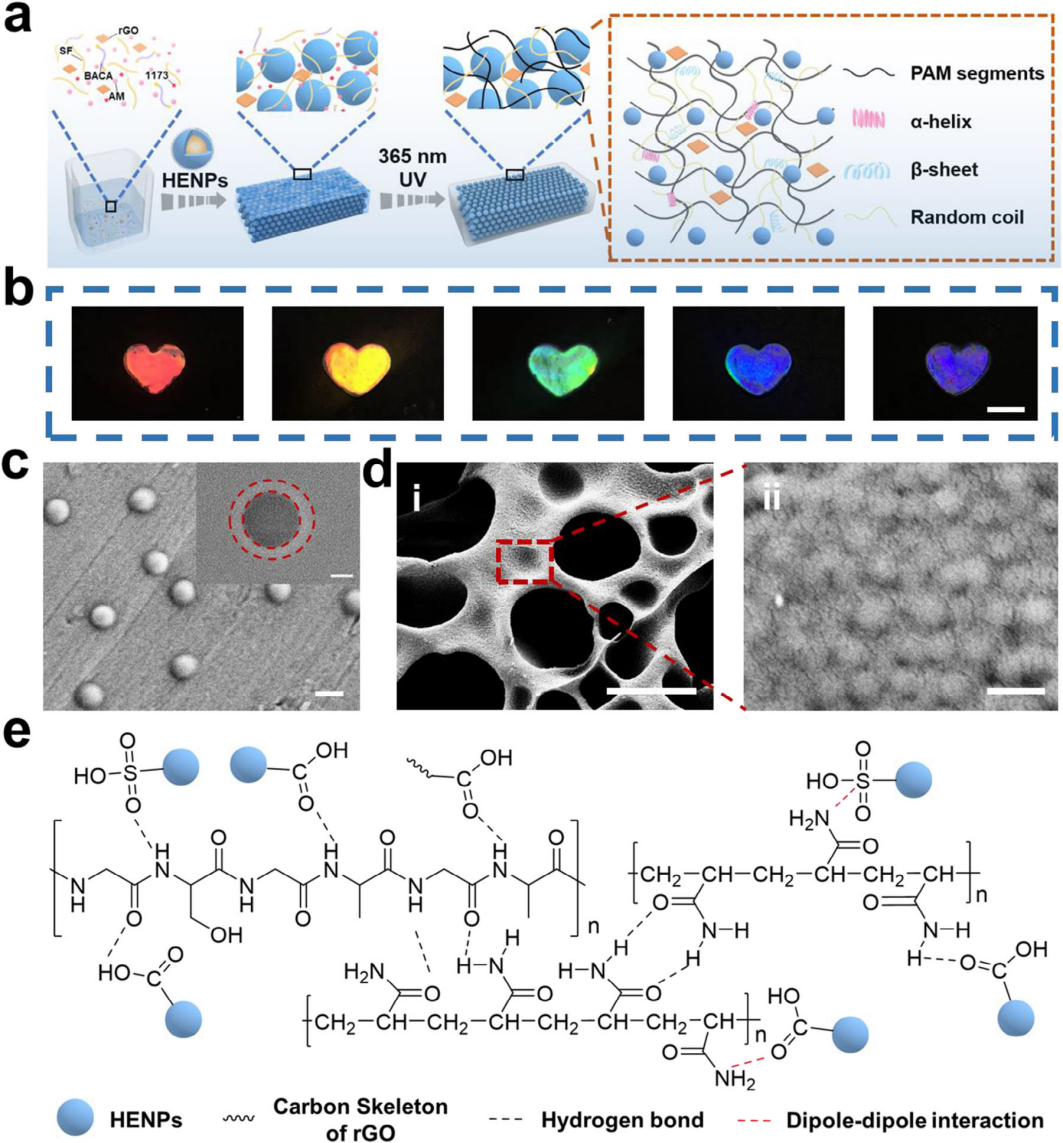
(a) Schematic illustration of the preparation of CSCH. (b) Photographs of CSCH with different structural colors. Scale bar is 1 cm. (c) SEM image of several separated HENPs. Scale bars is 200 nm. Inset on the top‐right corner, TEM image of the single HENPs. Scale bars is 50 nm. (d) SEM images of a lyophilized hydrogel sample. Scale bars are 10 µm and 500 nm for (i) and (ii), respectively. (e) Chemical structural formulas of dynamic bonds and dipole–dipole interaction in CSCH.

Figure 1a illustrates the schematic illustration of the fabrication procedures of CSCH. The CSCH was prepared by applying a simple ‘one‐pot’ assembly and photopolymerization procedure. Highly charged elastic nanoparticles were dispersed in a mixture comprised of acrylamide, SF, rGO, cross‐linkingagent *N*, *N*′‐bis(acryloyl)cystamine, and photoinitiator 2‐hydroxy‐2‐methylpropiophenone, and self‐assembled to form a non‐close‐packed array by interparticle repulsion. The dispersion was photopolymerized to produce the CSCH (named as PAM/SF/NP/rGO), which has a covalently cross‐linked network structure that embedded the non‐close‐packed HENPs array.

Highly charged elastic nanoparticles with a large number of –COOH and sulfonic acid groups on their surface endowed colloidal nanoparticles with high chargeability, [11] which enabled the elastic nanoparticles to self‐assemble and form a periodic photonic crystals structure. In addition, these elastic nanoparticles improve interfacial compatibility with flexible hydrogel networks. The covalently cross‐linked network structure and improved interfacial compatibility between HENPs and hydrogel endow the hydrogel with excellent mechanical properties.

PAM/SF/NP/rGO that covered the entire visible region from red to violet can be obtained easily by modulating the nanoparticle size (Figure 1b, Figure S1, Supporting Information). rGO as an additive can endow the hydrogel with high conductivity and increase the color contrast and the brilliant color of the hydrogel. As shown in the SEM and TEM images (Figure 1c), HENPs have a shell thickness of 18 nm and a core diameter of 120 nm. The SEM image for the PAM/SF/NP/rGO showed the existence of a 3D interconnected porous network with HENPs arranged periodically within it (Figure 1d). The periodic arrangement of HENPs endows PAM/SF/NP/rGO with characteristic reflection peaks and striking structural color due to the refraction and interference of the light in the periodic structure.

Due to the existence of the abundant reactive groups in hydrogel structure, there were many non‐covalent dynamic bond interactions, including the hydrogen bonds between PAM and SF, hydrogen bonds between PAM and HENPs, hydrogen bonds between SF and HENPs, and the dipole–dipole interaction between amide groups and sulfonic as well as carboxyl groups (Figure 1e). The integration of rGO also strengthens the hydrogen bonding with the various components. These noncovalent bond interactions endow the hydrogel with excellent self‐adhesiveness and healing properties.

**FIGURE 2:**
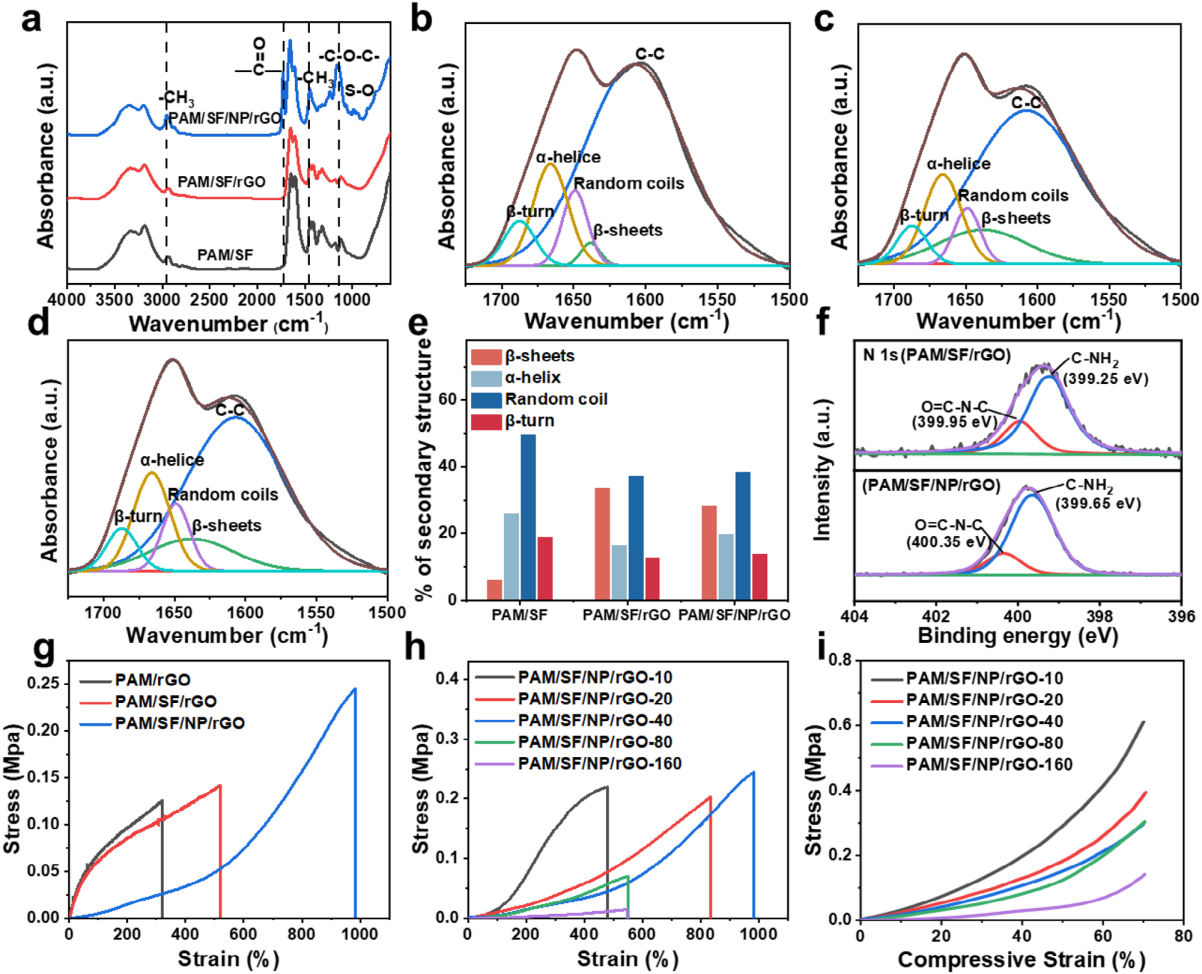
(a) FTIR spectra for PAM/SF, PAM/SF/rGO and PAM/SF/NP/rGO. (b–d) The deconvolution of FTIR spectra in amide I band of PAM/SF, PAM/SF/rGO and PAM/SF/NP/rGO. (e) Content of secondary structures in PAM/SF, PAM/SF/rGO and PAM/SF/NP/rGO obtained according to the amide I band in FTIR spectra. (f) XPS spectra of N1s for PAM/SF/rGO and PAM/SF/NP/rGO. (g) Tensile stress–strain curves of PAM/rGO, PAM/SF/rGO and PAM/SF/NP/rGO. (h) Tensile stress–strain curves and (i) compressive stress–strain curves of PAM/SF/NP/rGO with different content of SF (10, 20, 40, 80 and 160 µL).

The Fourier‐transform infrared (FT‐IR) spectroscopy of PAM/SF/NP/rGO showed characteristic bands at 1730 and 1060 cm^−1^ (Figure 2a), corresponding to the C═O stretching and S─O telescopic vibration peaks, respectively, indicating the successful introduction of HENPs [12]. The interface of graphene can induce conformational changes of SF from random coils and α‐helices to crystalline β‐structures through surface‐facilitated polypeptide chain folding [13]. These β‐structures are further stacked to form nanocrystals through hydrophobic interactions and hydrogen bonding. Disruption of the nanocrystals requires stronger forces and higher mechanical energy, and hydrogen bonding can be reorganized during stick‐slip deformation. Therefore, the β‐sheets and β‐turn structures of SF are critical for improving the mechanical properties of the samples. Further analysis of the FT‐IR spectroscopy results for the freeze‐dried hydrogel in the 1500–1725 cm^−1^ region quantified the β‐structure content of SF (Figure 2b–d). The relative percentages of β‐sheets, random coils, α‐helices, and β‐turn structures are shown in Figure 2e, revealing that the addition of rGO increases the proportions of β‐sheets and β‐turn structures in PAM/SF/rGO (together 46%) and PAM/SF/NP/rGO (42%), compared to pristine PAM/SF (25%).

Figure 2f displays the high‐resolution N 1s XPS spectra for PAM/SF/NP/rGO and PAM/SF/rGO hydrogel without elastic nanoparticles. The N 1s spectra of PAM/SF/rGO hydrogel exhibited two characteristic peaks of C‐NH_2_ (399.25 eV) and O═C─N─C (399.35 eV), while the peaks of PAM/SF/NP/rGO shifted to higher binding energy (from 399.25 to 399.65 eV, and 399.95 to 400.35 eV, respectively). These results indicated the strong interaction between the amino group of the hydrogel network and the carboxyl group, and the sulfonic acid of HENPs, which also demonstrated the significantly improved interfacial interactions between nanoparticles and hydrogel networks. This effect could be attributed to the hydrophilic and elastic shells of HENPs, which can improve their affinity for the hydrogel matrix and inhibit phase separation [14].

### 2.2 Mechanical Properties

The tensile stress–strain curves of the PAM/SF/rGO showed that the breaking elongation and tensile strength can reach up to 517% and 0.14 MPa, respectively (Figure 2g), which was higher than that of the PAM/rGO (about 318% and 0.12 MPa). This result indicated the high mechanical strength and strong stretching properties of the PAM/SF/rGO due to the addition of SF. This result could be ascribed to the formed interpenetrating network and β‐structure of SF, which was consistent with FT‐IR results. Meanwhile, the PAM/SF/NP/rGO hydrogel exhibited maximum breaking strain value and tensile strength, reaching up to 950% and 0.25 MPa, respectively, which was higher than that of the PAM/SF/rGO and completely met the requirement for human skin (<100 kPa). Meanwhile, as the content of HENPs increased, the breaking strain of the hydrogel increased significantly, but tensile stress decreased due to reduced density in the polymer network (Figure S2, Supporting Information). These results also indicated that the ordered nanoparticle array improved the mechanical strength of hydrogel. The reversible disruption and recombination of multiple dynamic non‐covalent cross‐linked networks serve to consume energy. In particular, the mechanical properties of the PAM/SF/NP/rGO hydrogel were obviously superior to that of these recently reported conductive structural color hydrogels (Table 1). This outcome could be interpreted considering that elastic and hydrophilic nanoparticles can improve the affinity and interfacial interactions with the flexible hydrogel network, which was consistent with the XPS results.

**TABLE 1:** Comparison of PAM/SF/NP/rGO properties with other photonic composites.

Ref.	Maximum strain	Constructive strategies	Tensile strength	Adhesiveness	Self‐healing	Optical sensing	Electrical sensing
[9]	300%	Three‐step method	0.12 Mpa	Adhesion strength: ≈30 kPa	No	Red to blue (632–478 nm) Detection range: 0%–80%	Gauge factor: 2.31
[6a]	635%	Self‐assembly	2.8 Mpa	Not adhesive	No	Red to green (610–520 nm) Detection range: 0%–80%	Gauge factor: 1.1
[10a]	≈380%	Replicating templates	0.55 Mpa	Adhesive to skin	Self‐healable	Red to violet (647–433 nm) Detection range: 0%–60%	Yes
[10b]	≈800%	Replicating templates	0.15 Mpa	Adhesive to skin	No	Red to blue (625–442 nm) Detection range: 0%–135%	Gauge factor: 2.59
[18]	≈650%	Magnetic field‐induced assembly	0.35 Mpa	Not adhesive	No	Red to violet (680–430 nm) Detection range: 0%–230%	Gauge factor: 47.2 (0%–130%) 191.8 (130%–300%)
[19]	≈101%	Self‐assembly	0.37 Mpa	Not adhesive	No	Orange to blue (604–500 nm) Detection range: 0%–60%	Gauge factor: 1
[20]	≈225%	Magnetic field induced assembly	0.52 Mpa	Not adhesive	No	Red to violet (680–430 nm) Detection range: 0%–110%	Gauge factor: 1.75
[17]	≈590%	Replicating templates	0.14 Mpa	Not adhesive	No	Red to blue Detection range: 0%–90%	Yes
This work	950%	Self‐assembly	0.25 Mpa	Adhesion strength: ≈3 kPa	Self‐healable	Red to violet (643–437 nm) Detection range: 0%–160%	Gauge factor: 1.46 (0%–100%) 3.31 (100%–400%)

Moreover, the impact of the SF content on the mechanical properties of the PAM/SF/NP/rGO hydrogel was further evaluated. Figure 2h,i shows the tensile stress–strain and compressive stress–strain curves for hydrogels with different SF contents. As the SF content increased, the tensile stress and fracture toughness of the PAM/SF/NP/rGO hydrogel gradually increased, reaching a peak at 40% SF content (PAM/SF/NP/rGO‐40, Figure 2h, Figure S3, Supporting Information). When the SF content exceeds 40%, the elongation and the tensile strength obviously decrease. Furthermore, the compressive stress of hydrogel gradually decreased as SF content increased (Figure 2i). The PAM/SF/NP/rGO‐40 exhibited the best mechanical properties with a maximum tensile strength (0.25 MPa), a maximum elongation at break (950%), a compressive stress over 0.3 MPa, and a Young's modulus of about 16.2 kPa. In addition, it is also noteworthy that our hydrogel exhibited excellent self‐recovery performance in cyclic tensile tests. No obvious shifting or breaking was observed after the implementation of 50 cycles of a cyclic tensile test at a strain of 100%, indicating the excellent mechanical sustainability of PAM/SF/NP/rGO (Figure S4, Supporting Information).

### 2.3 Adhesiveness Properties

**FIGURE 3:**
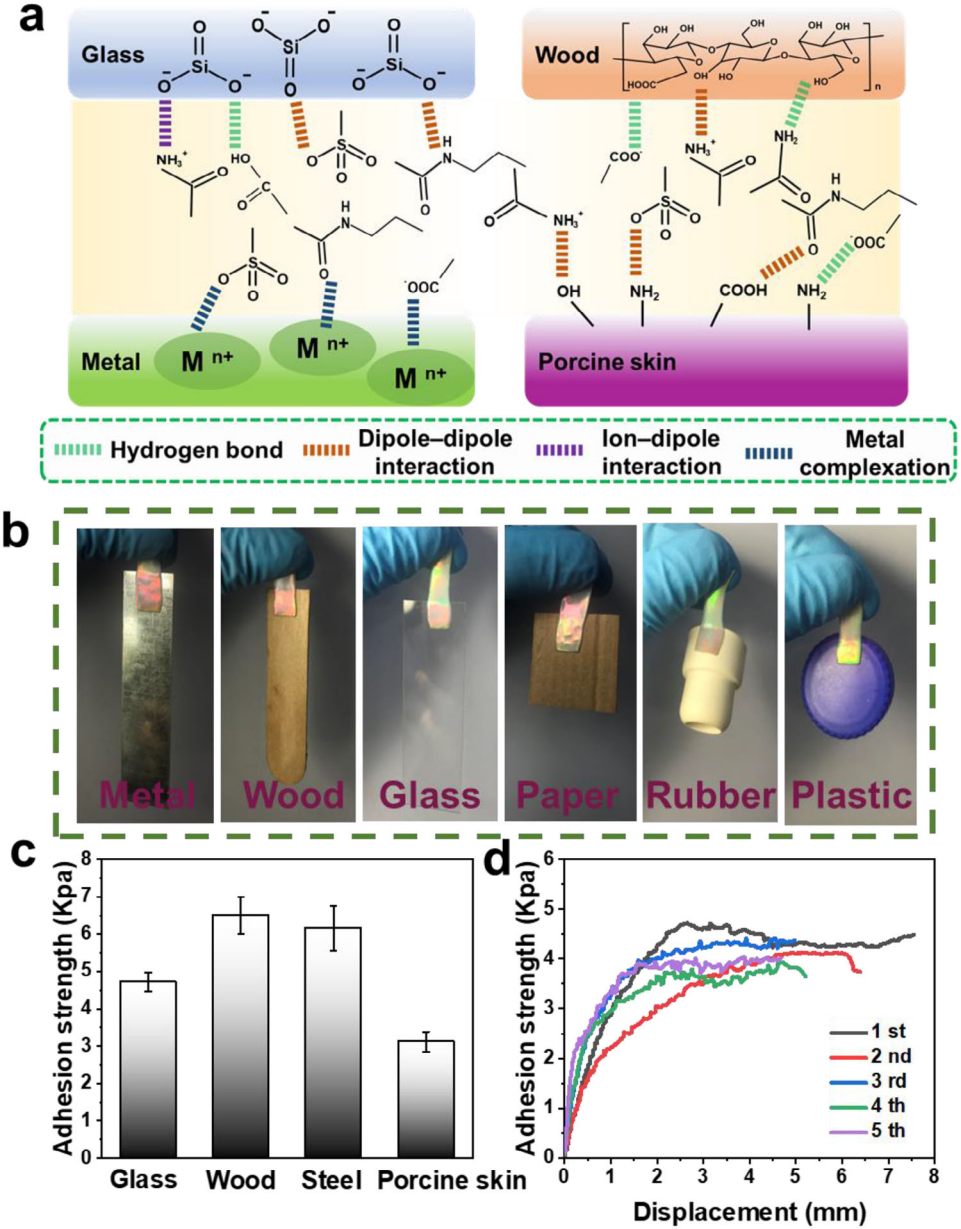
(a) Adhesion mechanism between PAM/SF/NP/rGO and various substrates. (b) Photographs of PAM/SF/NP/rGO adhering to different substrates (metal, wood, glass, paper, rubber, and plastic). (c) Adhesion strength on diverse substrates. (d) Adhesive strength to glass substrates with five cycling of adhesion and peeling.

The abundant polar amino, imino, and carbonyl functional groups in PAM/SF/NP/rGO can easily form multiple hydrogen bonds with the O, N, and F components of the substrate surface for adhesion, while the carbonyl group and sulfonic acid can generate metal coordination bonds with metal ions on the substrate surface for adhesion [15]. Moreover, sulfonic acid also contributes to adhesion through electrostatic interactions with the substrate. These synergetic interactions might simultaneously exist and endow the fabricated hydrogel with excellent adhesive capabilities (Figure 3a). Figure 3b shows that the hydrogel was firmly adhered to a variety of materials surfaces including metal, wood, glass, paper, rubber, and plastic can be achieved. The adhesion strength of the hydrogel was further quantified by standard lap shear tests (Figure S5, Supporting Information). As shown in Figure 3c, the adhesion strengths of our hydrogels were ≈4.7 KPa for glass, ≈6.5 KPa for wood, ≈6.2 KPa for steel, and ≈3.1 KPa for porcine skin. The reproducible adhesion behavior was also tested, and almost identical adhesion strength was observed over five cycles, indicating the excellent repeatable adhesion of our hydrogel. Remarkably, the proposed hydrogel could directly adhere to various irregular surfaces of the human skin, such as that of a finger, arm, and throat, and withstand the movement of the joint (Figure S6, Supporting Information), which is crucial for the development of interactive e‐skins. Furthermore, no obvious gel residue was observed on the skin after the application of several cycles of adhesion and peeling, while no signs of itching or redness were observed, confirming that our hydrogel meets the requirements for interactive e‐skin applications.

### 2.4 Self‐Healing Properties

**FIGURE 4:**
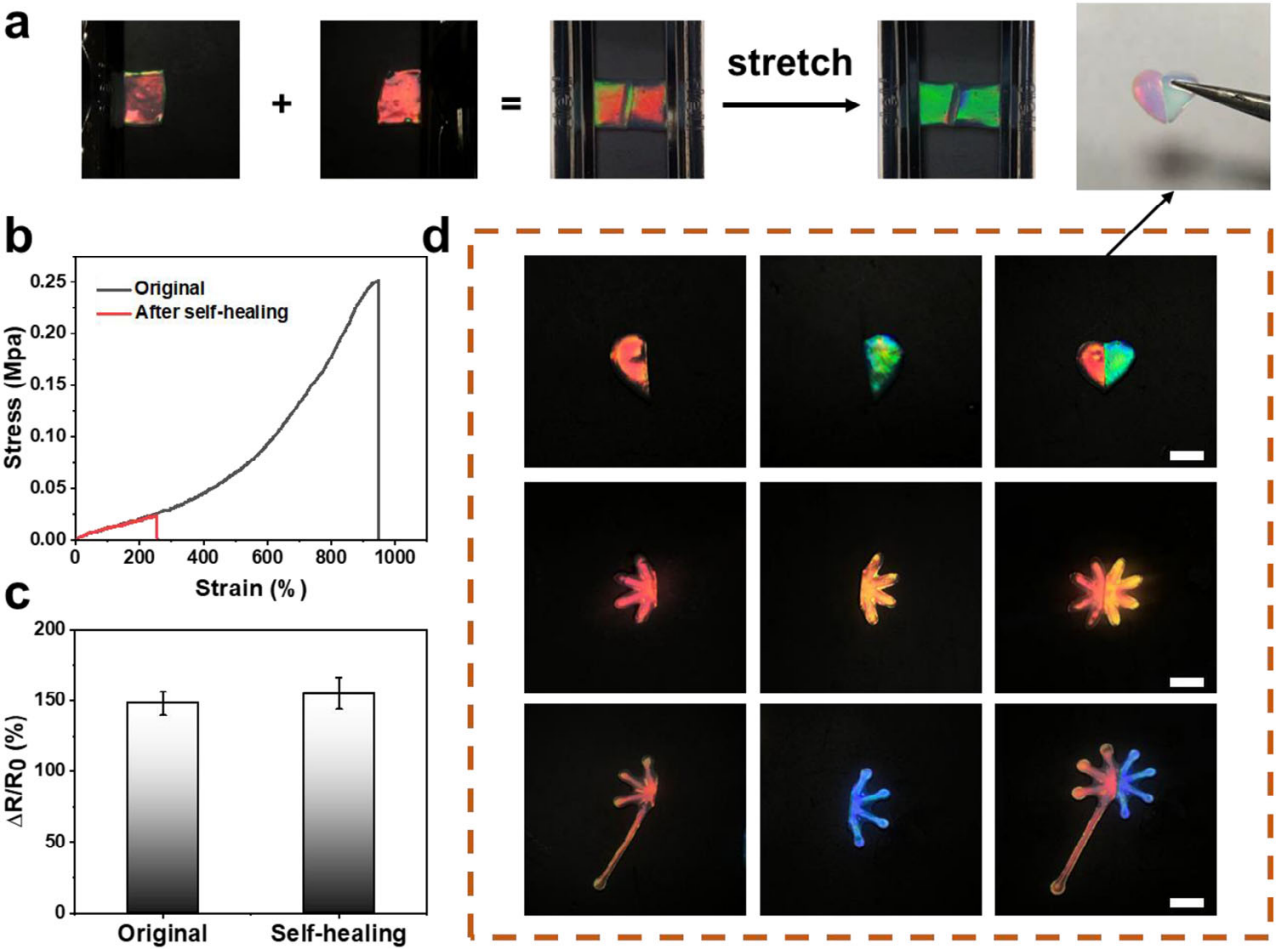
(a) Digital photographs showing the self‐healing property of PAM/SF/NP/rGO. (b) Stress–strain curves of the original and healed PAM/SF/NP/rGO. (c) The relative resistance changes of PAM/SF/NP/rGO before and after self‐healing at 100% strain. (d) Patterned self‐healed structural color film. Scale bar is 1 cm.

Due to the existence of various dynamic reversible hydrogen bonds, the developed hydrogel also exhibited excellent self‐healing capacity (Figure S7, Supporting Information). HENPs provide abundant noncovalent interactions with hydrogel networks, thus facilitating the recombination of hydrogen bonding and ionic bonding at the contact surface. To evaluate the self‐healing properties, the hydrogel was cut into two pieces that were contacted tightly and allowed to self‐heal at room temperature for 1 h. The cut hydrogel could be healed to form one segment and the healed hydrogel could withstand the large stretching deformations and displayed obvious color‐changing capacity under stretching (Figure 4a and Figure S8, Supporting Information). The tensile breaking rate of the healed hydrogel reached 250% and the healing efficiency of the mechanical properties was approximately 26.3%, calculated by comparing the tensile elongation ratio of the healed to the original hydrogel (Figure 4b). Although the tensile elongation of the healed hydrogel decreased, the tensile elongation of 250% was still well beyond human skin's stretching capacity. The decreased tensile strength of the healed hydrogel may be considered as being related to the characteristic that only noncovalent bonds between the two contact surfaces of the broken parts could be recovered again, while covalent bonds cannot. Furthermore, the relative resistance and conductivity of the hydrogel remained largely unchanged after healing (Figure 4c, Figure S9, Supporting Information), indicating that the fabricated hydrogel had excellent healing capability and the healing did not affect its conductivity, which is important for developing low‐cost hydrogel e‐skins.

Based on the excellent healing ability of the fabricated hydrogel, more complex and colorful patterns can be achieved. With the implementation of template molding methods, different patterns of hydrogels with various structural colors, such as heart, petals, and flowers, can be designed and fabricated by modulating the nanoparticle size. These hydrogels were then cut into two pieces and the different nanostructured segments with different structural colors could be healed into a whole at room temperature (Figure 4d). By employing such a self‐healing strategy, various complex patterns of hydrogels with multiple colors could be fabricated, which indicated the excellent healing ability of the proposed hydrogel and provided a simple and effective method for designing and manufacturing functional conductive hydrogel materials with various structural colors.

### 2.5 Synergistically Optical and Electrical Response Properties Under Strain

**FIGURE 5:**
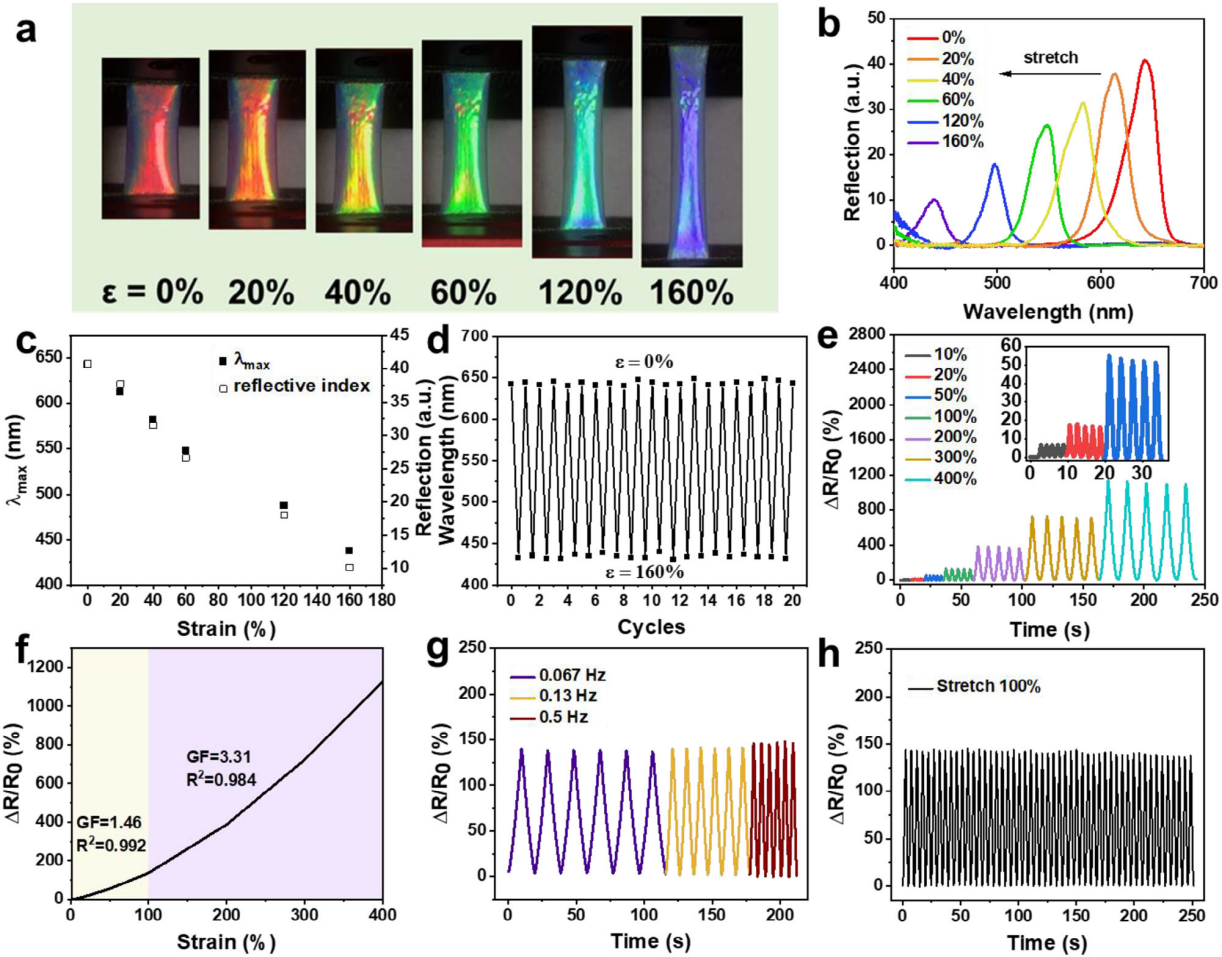
(a) Photographs and (b) corresponding reflection spectra of PAM/SF/NP/rGO under different strains. (c) The wavelength and the intensity of reflection peak under different strains. (d) Reversible change of *λ* max of PAM/SF/NP/rGO during stretching/releasing cycles at a strain of 160%. (e) Reversible response of the strain sensor during stretching–releasing cycles at different tensile strains from 10% to 400%. Inset: Zoomed‐in view for 10% to 50%. (f) Relative resistance as a function of strain under different strains. (g) Relative resistance changes of PAM/SF/NP/rGO under different stretching frequencies at 100% strain. (h) Relative resistance response under repeated stretching–releasing processes with a strain of 100%.

Benefiting from the ordered opal nanostructure, the as‐prepared conductive hydrogel can not only offer electrical signals but also provide synchronous and dynamic optical signals. The mechanical mechanochromic property of the as‐prepared hydrogel was investigated by stretching the film to different lengths (see Figure 5a). As the hydrogel was horizontally stretched from 0% to 160% strain, its structural color showed a distinct blue shift from its original red to violet. This result showed that the hydrogel had excellent stretchability and a broad optical sensing range of up to 160% strain, which could be attributed to the well‐established multiple dynamic bonding between the nanoparticles and the hydrogel network. Therefore, the high flexibility requirement of human skin could be met. In particular, the as‐prepared hydrogel had a broad optical sensing range of up to 160%, which was much higher than that of these recently reported conductive structural color hydrogels. This result showed that the application of the elastic nanoparticles as building units for structural color could improve the interfacial compatibility with the flexible hydrogel and its optical sensing range. The sharp structural color variation of PAM/SF/NP/rGO under different stretching strains also provided a simple naked‐eye visualization approach for visually detecting extensive stretching without any extra apparatus.

As can be observed in Figure 5b, the reflection peak of the as‐prepared hydrogel around the original wavelength of 643 nm also showed a continuous blue shift from 643 to 437 nm when stretched from 0% to 160%. A total wavelength change (Δ*λ*) of ≈206 nm is derived, which almost covers the complete visible range. This shift is due to the periodic arrangements of nanoparticles in the hydrogel matrix producing a photonic band gap and light of certain wavelengths located in the photonic band gap, which renders the propagation forbidden and thus the light is reflected selectively [16]. The corresponding reflection peak position could be determined according to Bragg's law [17]:

(1) λ=1.633dn,

where *λ* is the reflection peak wavelength, *d* denotes the center‐to‐center distance between the adjacent nanoparticles, and *n* represents the effective refractive index. When stretching the hydrogel, the thickness of the hydrogel decreased along with the strain direction, leading to the observed blue shift in the reflection peak according to Equation (1). Figure 5c shows the reflection peak position as a function of the tensile strain, where the blue shift of the reflection peak increases almost linearly with the increased strain, while the intensity of the reflection peak decreases with the increasing stretching strain. This relationship can be used to quantitatively analyze the stretching strain behavior.

The as‐prepared hydrogels showed excellent mechanical stability and full reversibility upon removal of extensional strain. When the extensional strain was removed, the hydrogel swiftly returned to its original color without any residual strain, which can be attributed to our hydrogel's excellent elasticity (Movie S1, Supporting Information). The reflection peak of the hydrogels fully recovered to the original position and no obvious loss of optical intensity and quality was observed after multiple strain cycles (Figure 5d). Hence, this result demonstrated the excellent recoverability and reliability of the as‐prepared hydrogels for practical e‐skin applications.

Synergistically electrical response performance of the as‐prepared hydrogels under strain was evaluated by measuring changes in their normalized resistance (Δ*R*/*R*_0_). As shown in Figure 5e, the normalized resistance of the hydrogel significantly increased as the stretching strain was amplified from 10% to 400%. This result shows that the hydrogel sensor could be used for quantitative analysis of the applied stretching strain. The GF of the hydrogel sensor was calculated to determine the sensor's sensitivity. As shown in Figure 5f, the GF of the proposed hydrogel was approximately 1.46 for tensile strains ranging from 10% to 100% and increased to 3.31 for strains between 100% and 400%. The largest GF value for the hydrogel sensor was about 3.31, which was clearly higher than that of the recently reported other conductive structural color hydrogel sensors, demonstrating superior sensitivity. Furthermore, the hydrogel sensor showed an immediate response time of about 420 ms under 100% stretching strain with a rate of 50 mm s^−1^, and the response time was sufficient enough to be used for e‐skins (Figure S10, Supporting Information).

The hydrogel sensor exhibited excellent stable and reproducible electromechanical response under repeated stretching–releasing cycles not only with different strain loads ranging from 10% to 400%, but also with different stretching frequencies (Figure 5g). Furthermore, multiple stretching–relaxing tests with 100% strain were applied on the hydrogel sensor and were followed by a full recovery to the relaxed state. The resistance of the hydrogel sensor measured under each stretching and relaxed state all exhibited almost identical changes within 100 cycles (Figure 5h), demonstrating that the sensor has great reliability and mechanical recovery.

### 2.6 Applications as an Interactive E‐Skin

**FIGURE 6:**
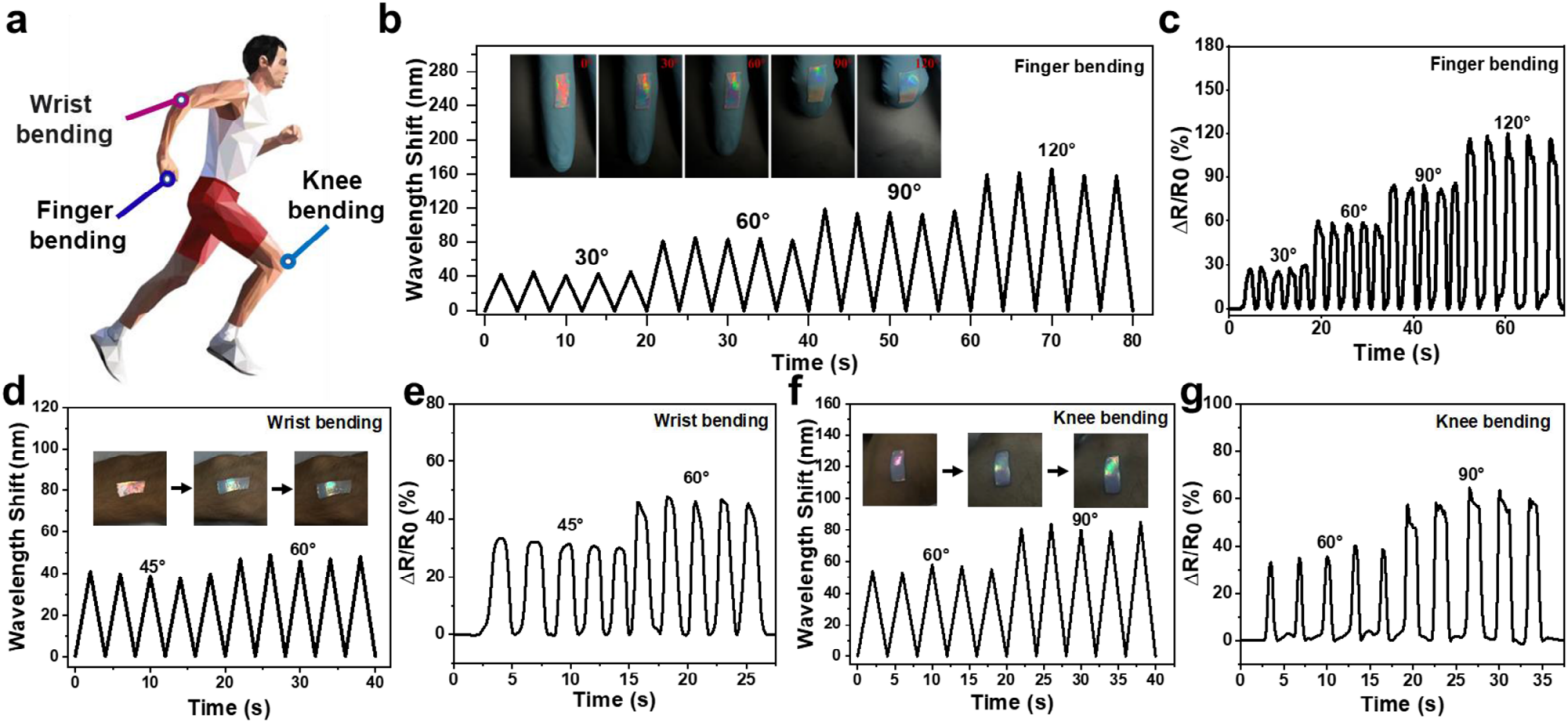
(a) Schematic illustration of different joint movements of the human body. Real photographs and corresponding reflectance spectra of the as‐prepared PAM/SF/NP/rGO when monitoring joint movements of (b) finger, (d) wrist, and (f) knee. (c,e,g) Corresponding signals of relative electrical resistance.

Having established their superior performance, the PAM/SF/NP/rGO could be used as an interactive e‐skin for synchronous electronic and visual signal monitoring of various body movements. As shown in Figure 6a, the hydrogel sensors could be directly adhered to the finger, wrist, and knee for real‐time monitoring of human joint motion. When the finger‐bending angles gradually increased, the hydrogel sensor displayed remarkable color changes from red to blue‐green with an obvious blue shift in the reflection peak (Figure 6b). Moreover, the reflection peak and the structure color of the hydrogel appeared excellent recovery performance and reproducibility even after suffering from the continuous bending‐stretching motion of the finger. These results demonstrated that the different degrees of finger joint movement could be detected and distinguished through the change of the simple color or the reflection peak of the sensor.

At the same time, the resistance of the hydrogel sensor exhibited an immediate synchronous increase with the increased bending angles of the finger while the output electrical signals showed good repeatability and stability (Figure 6c and Figure S11, Supporting Information). Furthermore, the hydrogel sensor also displayed stable dual‐signal responses when it was adhered onto the wrist and knee (Figure 6d–g). The structural color and resistance in the sensor all exhibited rapid and stable changes according to the deformations caused by wrist and knee bending. More attractively, benefiting from its one‐layer covalent cross‐linking network structure and excellent adhesion, the hydrogel sensor did not delaminate and detach from the skin even during extensive joint movements. All these results indicated that the proposed hydrogel sensor has an interactive color‐changing ability and sensitive electronic responses in dynamic activities, which displays promising applications in interactive e‐skins for motion and health monitoring, rehabilitation medicine, soft robots, and so on.

**FIGURE 7:**
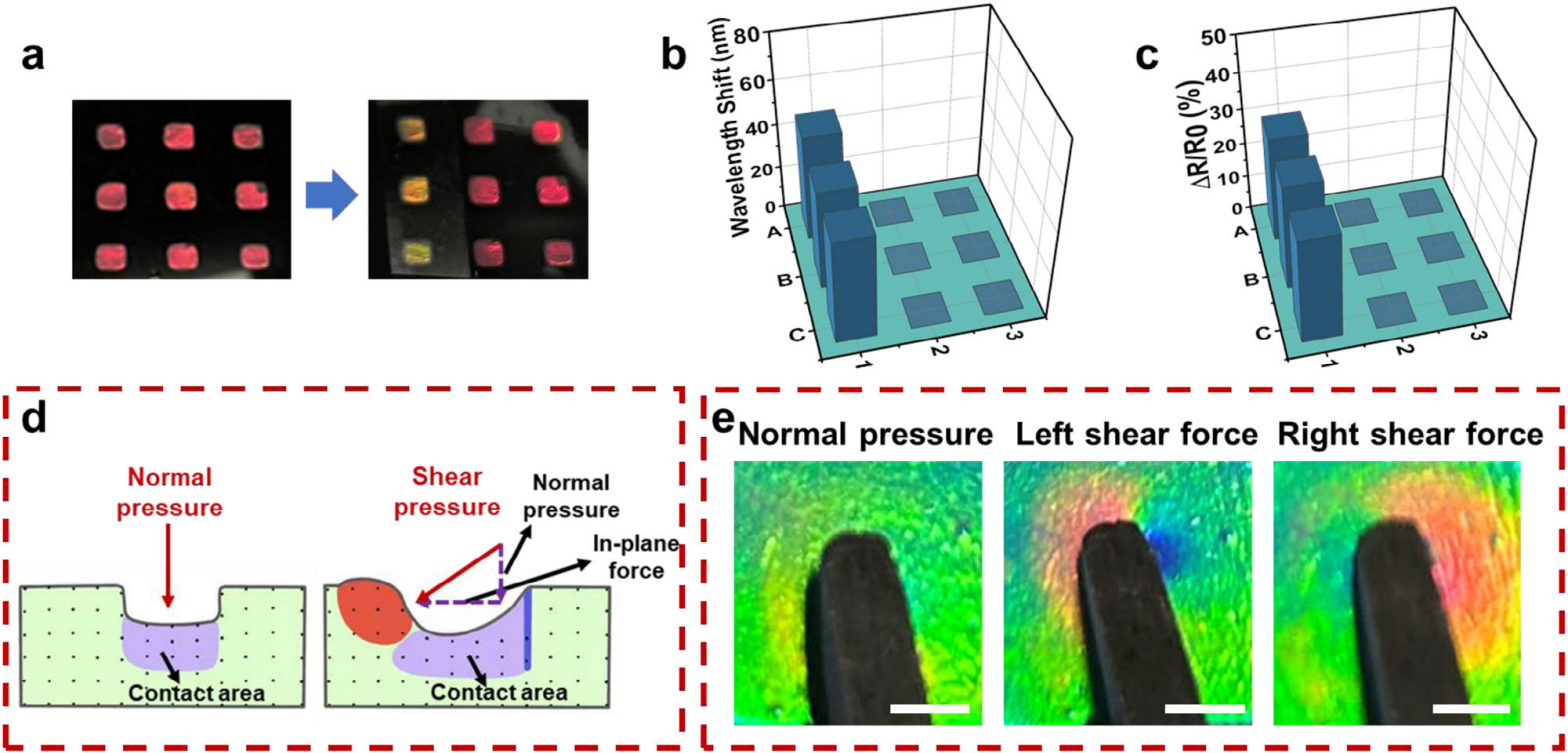
(a) Real photographs of the 3 × 3 hydrogel array consisting of rectangular sensing pixels (pixel size, 0.8 cm × 0.8 cm, pixel spacing, 1 cm) and distribution of the structure color change of the sensor array during loading a transparent glass sheet on the cells labeled A1, B1 and C1. (b,c) The distribution of the corresponding reflection peak and relative electrical resistance change of the sensor array. (d) Schematic illustration of the thickness direction of PAM/SF/NP/rGO under normal pressure and shear force. (e) Photographs of corresponding structural color distributions of the hydrogel. Scale bar is 1 cm.

Based on a simple one‐step assembly and photopolymerization method, a 3 × 3 array of the as‐prepared hydrogel was prepared with a mold for sense spatial distribution of pressure or touch force. As shown in Figure 7a, when a transparent glass sheet was placed on the cells labeled A1, B1, and C1, the structure color in these specific cells showed a distinct blue shift from red to orange while no obvious change was observed in other cells. The results indicated that the color information in our hydrogel array successfully reflected the real‐time pressure distribution. Therefore, this array provided a simple naked‐eye visualization approach to detecting spatial pressure distribution without any extra apparatuses. Furthermore, the sensor unit under the glass sheet showed an obviously increased reflection peak shift and electrical resistance because of pressure stimuli (Figure 7a–c and Figure S12, Supporting Information), whereas the remaining units in the array did not respond. The above‐mentioned results clearly indicated that our hydrogel array could identify the spatial pressure distribution qualitatively and quantitatively by their color signals and electrical signals, respectively.

It is difficult to characterize different types of external pressure, such as normal pressure and additional in‐plane compression/stretches using electrical signals (Figure 7c). However, the optical signal output provided by the introduction of 3D photonic crystal offers significant advantages in detecting and distinguishing external pressure stimuli. Due to the excellent mechanochromic properties of PAM/SF/NP/rGO, the spatial stress distribution in the area adjacent to the contact point generated by the pressure stimulus could be translated into a visual color map. When PAM/SF/NP/rGO was subjected to different external stimuli, such as normal pressure and shear force, the visual signals could effectively distinguish stress fields caused by 3D deformation. As shown in Figure 7d, almost only the material below the contact area was deformed when subjected to normal pressure, while the in‐plane compression generated by shear forces pushed the material bulge immediately in front of the contact point causing a structural color red shift, whereas the area behind the contact point exhibited a slight blue shift due to stretching (Figure 7e and Figure S13, Supporting Information). Thus, the visual color map effectively identified the location and type of stress, as well as the direction of the shear force. Moreover, the PAM/SF/NP/rGO could visually identify the shape and location of complex spatially distributed pressure sources, such as fingerprints (Figure S14, Supporting Information). According to the above‐mentioned results, it can be inferred that PAM/SF/NP/rGO has exciting potential for applications in interactive sensing.

## 3 Conclusion

In summary, a conductive structural color hydrogel with a one‐layer structure was developed via a simple one‐step assembly and photopolymerization strategy. The resulting hydrogel achieved outstanding excellent mechanical robustness, self‐adhesiveness, self‐healing properties as well as synchronous electronic and visual signal monitoring through ingenious structural design. Especially, the HENPs were elaborately applied as building units for structural color and improved the interfacial compatibility for effective energy dissipation. Moreover, the ordered nanoparticle array containing a large number of ─COOH and sulfonic acid groups enrich the non‐covalent interactions of the hydrogel, resulting in excellent self‐adhesiveness and self‐healing properties. Compared with many reported conductive structural color hydrogels in the literature (Table 1), our material shows a unique combination of advantages in terms of multiple physicochemical properties, including mechanical properties, self‐adhesiveness, self‐healing ability, optical sensing range, and gauge factor. The proposed hydrogel can be used as an interactive e‐skin for color‐response and electrical signal response of various human motions, the spatial distribution of external mechanical stimuli, as well as identification of different external stimuli. As a result, it is expected to provide a sustainable, durable and transient platform for the development of fields interactively visual e‐skin, wearable devices, and bionics, among others.

## 4 Experimental Section

The experimental section is available in the supporting information of the manuscript. All human sensing demonstrations were conducted at Southeast University (China), with subjects having signed informed consent forms prior to participation.

## Conflicts of Interest Statement

The authors declare no conflicts of interest.

## Supporting information



Supporting Information

Supporting Information

## Data Availability

The data that support the findings of this study are available from the corresponding author upon reasonable request.
