# Supplementary material for: Elastic Nanoparticle‐Reinforced, Conductive Structural Color Hydrogel With Super Stretchability, Self‐Adhesion, Self‐Healing as Electrical/Optical Dual‐Responsive Visual Electronic Skins
Source: Exploration (Beijing). 2025 Feb 4;5(2):270008. doi: 10.1002/EXP.70008 (PMC12087393; doi:10.1002/EXP.70008)
Supplement: Supplementary file 1 — Supporting Information [file EXP2-5-270008-s001.docx]

**Supplementary Material**

**Elastic Nanoparticle-Reinforced, Conductive** **Structural Color Hydrogel with Super Stretchability, Self-Adhesion, Self-Healing as Electrical/Optical Dual-****responsive Visual Electronic Skins**

*Min Xu^1^,* *Junlong Liao^1^, Jiajia Li^1^, Yu Shi^1^, Ziyu Zhang^1^, Yifu Fu^1^, Zhongze Gu**^1,2^,* and Hua Xu^1,2^**

^1^State Key Laboratory of Digital Medical Engineering, School of Biological Science and Medical Engineering, Southeast University, Si Pai Lou 2, Nanjing 210096, China

^2^Institute of Biomedical Devices (Suzhou), Southeast University, Suzhou 215163, Jiangsu, China

E-mail: gu@seu.edu.cn; [huaxu@seu.edu.cn](mailto:huaxu@seu.edu.cn)

**Experimental Section**

**Materials**

Acrylic acid (AA), acrylamide (AM), methyl methacrylate (MMA), n-butyl acrylate (BA), sodium p-styrene sulfonate (NaPSS), ammonium persulfate (APS), ethylene glycol dimethacrylate (EGDMA), silk fibroin (SF), *N*,*N*′-bis(acryloyl)cystamine (BACA), and 2-hydroxy-2-methylpropiophenone (1173) were obtained from Sigma-Aldrich. rGO was purchased from Nanjing XF NANO Materials Tech Co., Ltd. Ion-exchange resin (AG501-X8(D)) was obtained from Bio-Rad (USA). All chemicals were used as received. Deionized water with a resistivity of 18.2 MΩ·cm was used for the preparation of all aqueous solutions.

**Fabrication of HENPs**

The copolymer nanoparticles were synthesized by emulsifier-free emulsion polymerization. Briefly, 3 mL of MMA, 3 mL of BA, 50 µL of EGDMA, 400 µL of AA and 60 mL of deionized water were added to a 100 mL three-necked flask, and the mixture was stirred at 80°C under nitrogen atmosphere for 0.5 h. Afterward, 4 mL of an aqueous solution containing 180 mg of APS, desired amount of NaPSS was added into the mixture. The reaction lasted for 8 h. The obtained products were collected by centrifugation and washed three times with deionized water.

**Construction of** **CSCH**

CSCH were synthesized by the one-pot method. First of all, HENPs was added to deionized water (300 µL) containing AM (150 mg), BACA (1.5 mg), SF solution (10 µL,10 vol%), rGO (50 µL, 0.5 mg mL^−1^), and HMPP (3 µL). The precursor subsequently was infiltrated into a gap between two glass slides separated by 500-µm-thick spacers of polyimide tape (Kapton) by capillary force, following the polymerization exposed to UV light for 2 min. Before the HENPs are dispersed in the precursor solution, an appropriate amount of ion exchange resin should be added into the precursor solution to remove free impurity ions. Afterwards, the ion exchange resin should be separated. Following the same process except replacing the HENPs with deionized water, we prepare the PAM/SF/rGO as control groups. In the above process, PAM/SF and PAM/rGO were obtained by replacing rGO or SF solution with aqueous solution, respectively, and the rest was the same.

**Construction of Patterned CSCH**

By using mask templates, different patterned hydrogel with various structural colors were fabricated. Different colors patterned hydrogel segments were chosen and assembled together for 1 h. The structural color of our hybrid materials can be observed clearly by naked eye under normal white light. Additional light illumination was only used for take the bright pictures of the materials.

**Characterization**

FTIR tests were carried out on a Nicolet IS10 spectrometer (Thermo Fisher Scientific) in the wavelength range from 1000 to 4000 cm−1. SEM images were taken by using a field emission scanning electron microscope (FESEM, Zeiss Ultra Plus). Transmission electron microscopy (JEM-2100EX) was used to observe the structure of the HENPs. Mechanical tests (tensile and compressive behaviors) were performed with a Microforce Tester (Instron 5943). Reflection spectra were performed by using a spectrophotometer (Ocean Optics, QE65000). The resistance of the samples was obtained with semiconductor characterization system (Keithley 4200-SCS). XPS spectra were obtained using an ESCALAB 250XIþ 5600 system from Thermo Scientific.


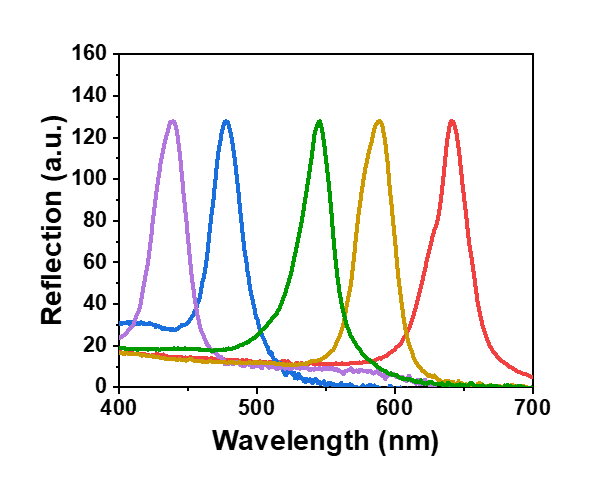


**Figure S1.** Reflectance spectra of PAM/SF/NP/rGO prepared from HENPs with diameters of 113, 125, 138, 155 and 192 nm (from left to right).


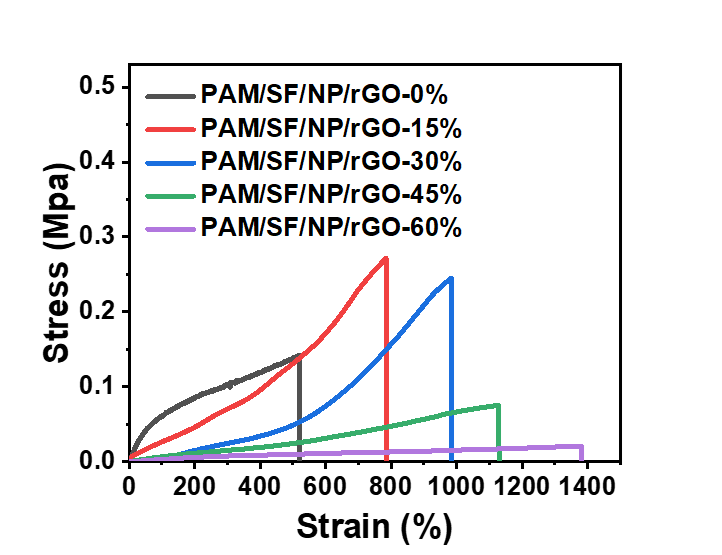


**Figure S2.** Stress-strain curves of PAM/SF/NP/rGO with various content of HENPs (0 wt%, 15 wt%, 30 wt%, 45 wt% and 60 wt%).


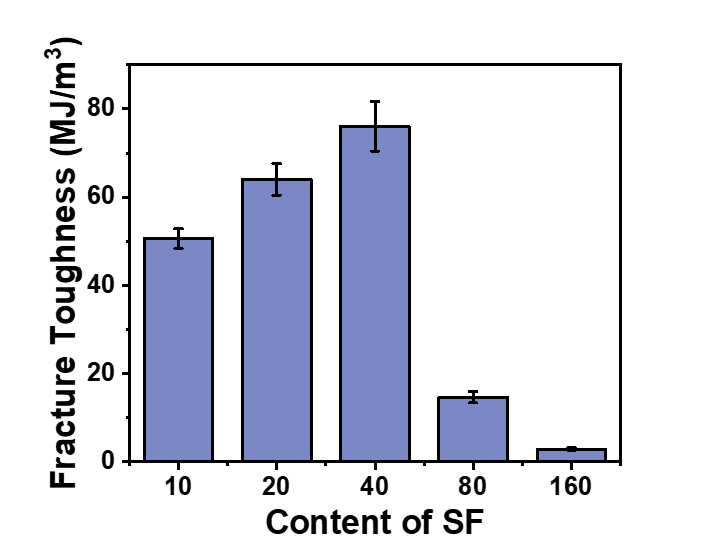


**Figure S3.** Fracture toughness of PAM/SF/NP/rGO with different content of SF (10, 20, 40, 80 and 160 µL).


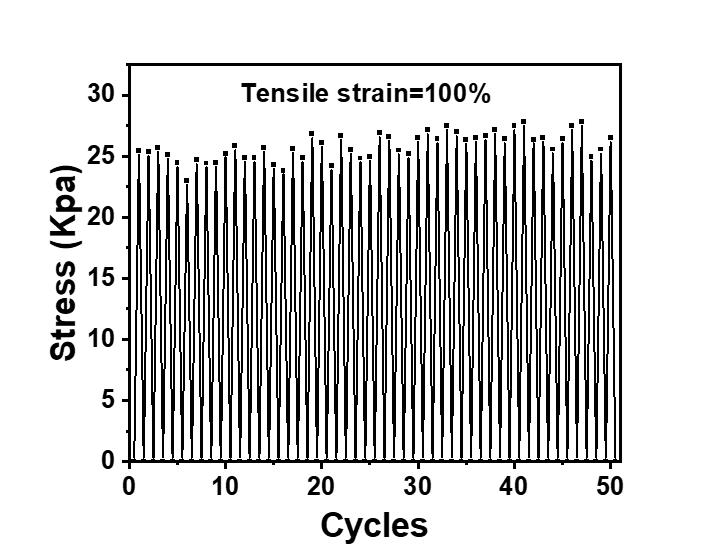


**Figure S4.** Tensile cycles of PAM/SF/NP/rGO at a strain of 100%.


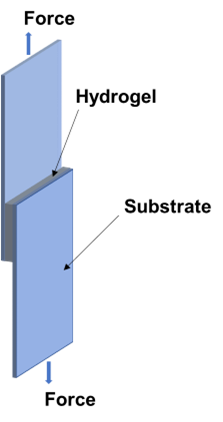


**Figure S5.** Schematic description of the lap shear test.

**
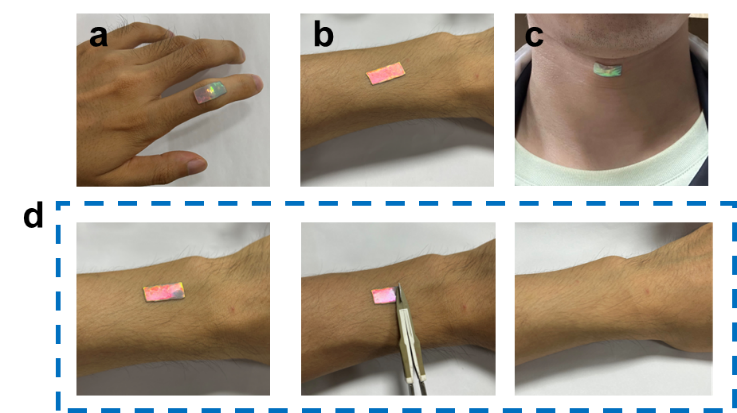
**

**Figure S6.** a, b, c) Photographs of PAM/SF/NP/rGO sticking to uneven surface (finger, arm, throat). d) Exhibition of stripping lag and no residual behavior in the process of peeling from the human skin.


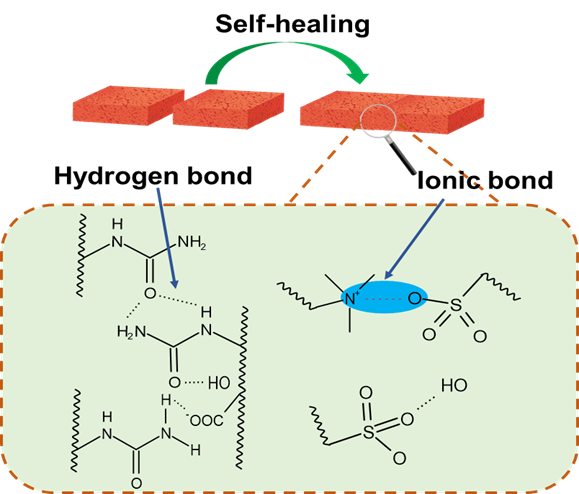


**Figure S7.** Schematic diagram of the self-healing mechanism of PAM/SF/NP/rGO.

**
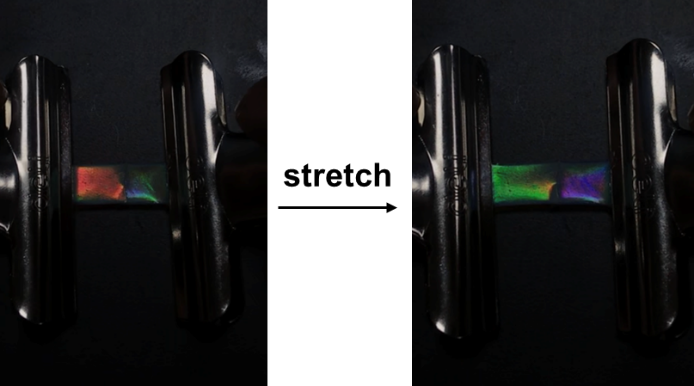
**

**Figure S8.** Photographs showing the self-healing property of PAM/SF/NP/rGO.


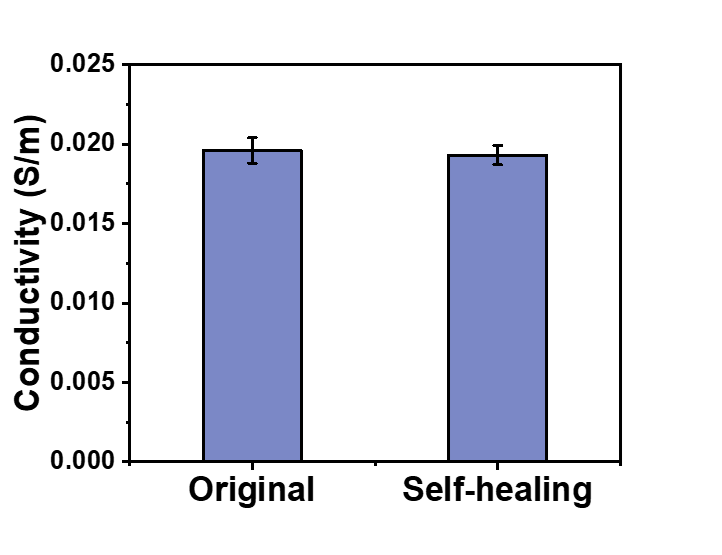


**Figure S9.** Conductivity of original and self-healing PAM/SF/NP/rGO.

**
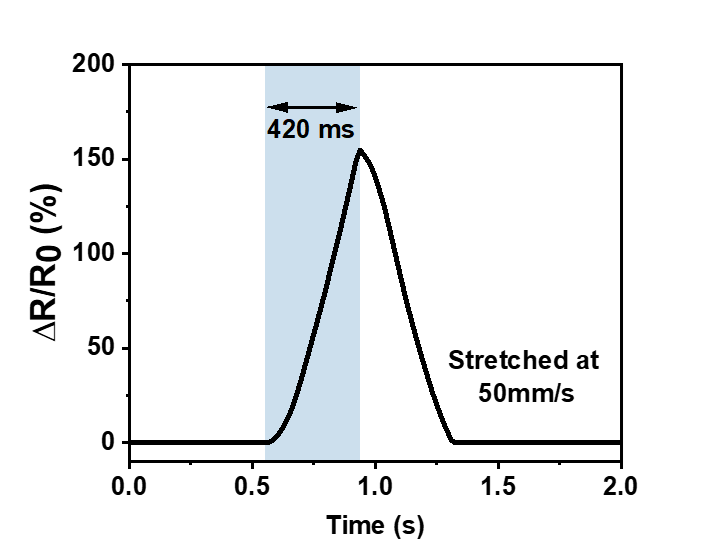
**

**Figure S10.** Response time of the relative resistance signal of PAM/SF/NP/rGO.


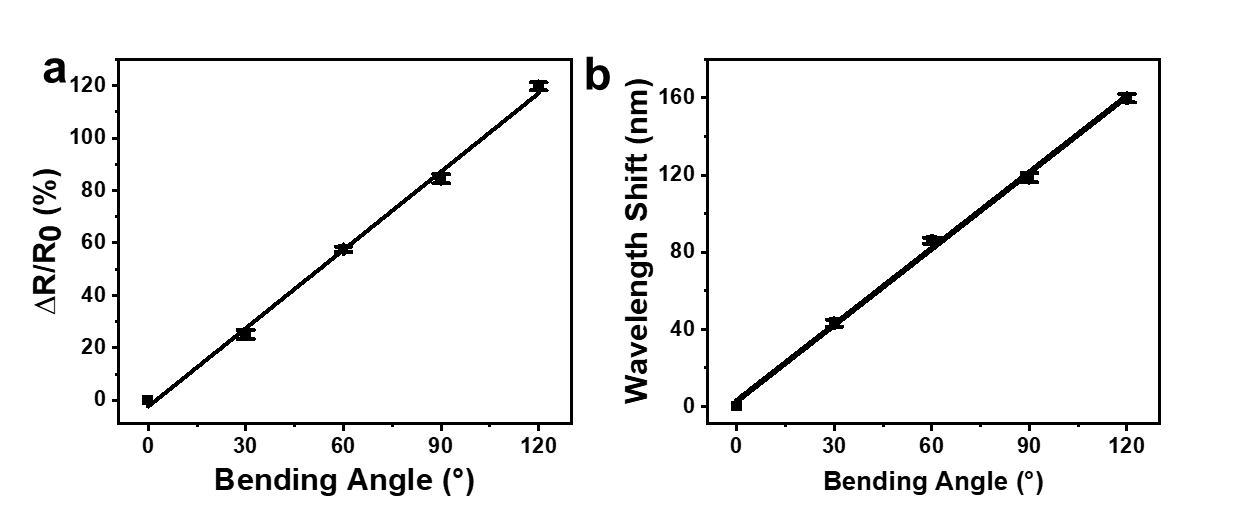


**Figure S11.** a) Relative resistance change of PAM/SF/NP/rGO in response to different finger bending angles. b) The wavelength shift values of PAM/SF/NP/rGO in response to finger bending angles.


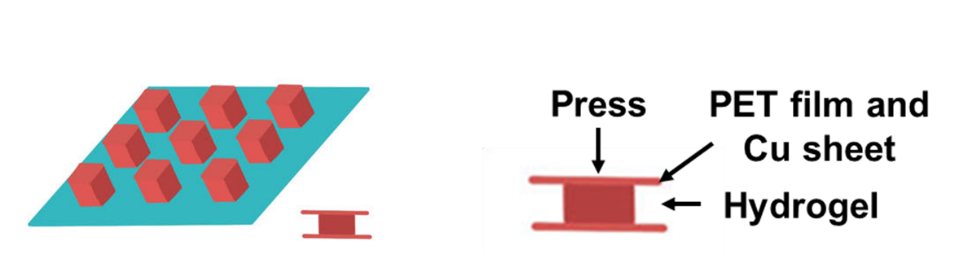


**Figure S12.** a) The 3 × 3 array of flexible sensors that hydrogel cubes connected with copper sheets.

**
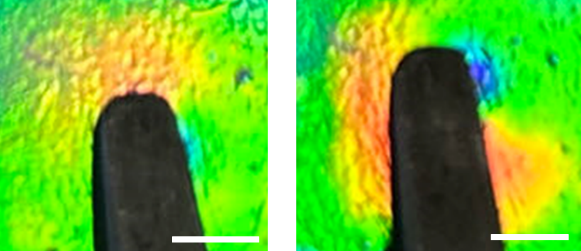
**

**Figure S13.** Photographs of structural color distribution under different directional shear forces. Scale bar is 1 cm.

**
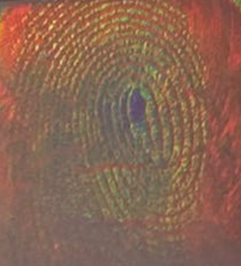
**

**Figure S14.** Photographs of structure color distribution when finger pressed.
